# Essential roles of C-type lectin Mincle in induction of neuropathic pain in mice

**DOI:** 10.1038/s41598-018-37318-8

**Published:** 2019-01-29

**Authors:** Asako Ishikawa, Yasunobu Miyake, Kimiko Kobayashi, Yuzo Murata, Sayaka Iizasa, Ei’ichi Iizasa, Sho Yamasaki, Naomi Hirakawa, Hiromitsu Hara, Hiroki Yoshida, Toshiharu Yasaka

**Affiliations:** 1 Department of Anesthesiology & Critical Care Medicine, Faculty of Medicine, Saga University, 5-1-1 Nabeshima, Saga, 849-8501 Japan; 2 Division of Molecular and Cellular Immunoscience, Department of Biomolecular Sciences, Faculty of Medicine, Saga University, 5-1-1 Nabeshima, Saga, 849-8501 Japan; 3 Department of Anatomy and Neuroscience, Hyogo College of Medicine, 1-1 Mukogawa-cho, Nishinomiya, Hyogo, 663-8501 Japan; 4 Division of Histology and Neuroanatomy, Department of Anatomy & Physiology, Faculty of Medicine, Saga University, 5-1-1 Nabeshima, Saga, 849-8501 Japan; 5 Department of Biological Science and Technology, The United Graduate School of Agricultural Sciences, Kagoshima University, 1-21-24 Korimoto, Kagoshima, 890-8580 Japan; 6 Department of Immunology, Graduate School of Medical and Dental Sciences, Kagoshima University, 8-35-1 Sakuragaoka, Kagoshima, 890-8544 Japan; 7 Division of Molecular Immunology, Medical Institute of Bioregulation, Kyushu University, 3-1-1 Maidashi, Higashi-ku, Fukuoka, 812-8582 Japan; 8 Pain Clinic & Palliative Care Medicine, Saga University Hospital, 5-1-1 Nabeshima, Saga, 849-8501 Japan

## Abstract

Increasing evidence indicates that pattern recognition receptors (PRRs) are involved in neuropathic pain after peripheral nerve injury (PNI). While a significant number of studies support an association between neuropathic pain and the innate immune response mediated through Toll-like receptors, a family of PRRs, the roles of other types of PRRs are largely unknown. In this study, we have focused on the macrophage-inducible C-type lectin (Mincle), a PRR allocated to the C-type lectin receptor family. Here, we show that Mincle is involved in neuropathic pain after PNI. *Mincle*-deficient mice showed impaired PNI-induced mechanical allodynia. After PNI, expression of *Mincle* mRNA was rapidly increased in the injured spinal nerve. Most *Mincle*-expressing cells were identified as infiltrating leucocytes, although the migration of leucocytes was also observed in *Mincle*-deficient mice. Furthermore, *Mincle*-deficiency affected the induction of genes, which are reported to contribute to neuropathic pain after PNI in the dorsal root ganglia and spinal dorsal horn. These results suggest that Mincle is involved in triggering sequential processes that lead to the pathogenesis of neuropathic pain.

## Introduction

Neuropathic pain results from damage to the peripheral nervous system (PNS) and/or the central nervous system (CNS), and is characterized by allodynia (pain evoked by a normally innocuous stimulus) and hyperalgesia (enhanced pain evoked by a noxious stimulus). Neuropathic pain remains a persistent clinical problem because of an incomplete understanding of the complex pathogenesis. Among such complex mechanisms underlying neuropathic pain, the neuroimmune interface has been reported to play an important role in inducing these hypersensitivities^1–3^. After peripheral nerve injury (PNI), various mediators such as cytokines, chemokines, and neurotransmitters, released from the damaged nerves, alter the responsiveness of various immune cells in the PNS and CNS^4^. Various immune cells are recruited to the injury site. These cells release numerous immune molecules such as cytokines and chemokines that can directly alter the nociceptive information^4^. Recently, the pattern recognition receptors (PRRs) have been receiving attention as molecules that induce pathological changes in chronic pain^5–7^. PRRs were originally classified as the types of receptors that sense exogenous pathogen-associated molecular patterns (PAMPs) and induce an innate immune response^8^. Now it is evident that endogenous molecules released from dying or damaged cells, referred to as damage-associated molecular patterns (DAMPs), also activate the PRRs during tissue injuries^9^. The ability of PRRs to be activated by DAMPs is particularly interesting because cells expressing PRRs can be activated in injured tissues and then can trigger reactions that lead to neuropathic pain under sterile conditions. Toll-like receptors (TLRs) are a well-studied family of PRRs and have been reported to play a significant role in innate immunity^8^. Notably, TLRs are expressed by numerous cell types in the CNS and PNS including both neuronal and non-neuronal cells such as the microglia (the resident macrophages in the CNS)^10,11^ and contribute to the induction and maintenance of neuropathic pain conditions^6,7^. While evidence regarding the roles of TLRs in neuropathic pain is accumulating, those of another type of PRRs, consisting of the C-type lectin receptors (CLRs), have not yet been investigated. Macrophage-inducible C-type lectin (Mincle), one of the CLRs, is expressed mainly in the myeloid cells and is induced after exposure to various stimuli and stress^12^. Mincle is known to recognize not only the PAMPs derived from mycobacterium and fungus^13,14^, but also the DAMPs derived from dead cells^15,16^, to induce inflammatory responses. In rodent models of cerebrovascular disorders, Mincle is reported to be expressed in microglia-like cells within damaged brain areas, and contributes to innate immune responses^17,18^. Therefore, we hypothesized that Mincle activation by DAMPs in damaged nerves could trigger the innate immune response and the following inflammatory events that would result in induction of neuropathic pain after PNI. Here, we show for the first time that Mincle is involved in pathological processes in neuropathic pain. *Mincle*-deficient mice showed impaired development of mechanical allodynia after PNI. Expression of *Mincle* mRNA was rapidly increased in the injured spinal nerve (SN) after PNI and most *Mincle*-expressing cells were identified as infiltrating neutrophils but not the dorsal root ganglion (DRG) neurons. Furthermore, the increase in *Mincle* mRNA expression and neutrophil migration in injured SN after PNI was found to depend on myeloid differentiation primary response gene 88 (MyD88), one of the major adaptor proteins of the TLR signalling pathways. Our results suggest that Mincle in the injured nerve induces neuropathic pain by triggering sequential processes that lead to tactile allodynia in the DRG and spinal dorsal horn.

## Results

### Mincle contributes to the mechanical hypersensitivity after peripheral nerve injury

Mice lacking the Mincle gene (*Mincle*^−/−^) appeared healthy and showed no obvious abnormality on visual inspection^14^. Before assessing pain behaviour, we examined the locomotor activity and anxiety of *Mincle*^−/−^ mice because these factors are known to influence the reliability of assessment of pain behaviours. The open-field test was used to assess anxiety-like behaviour and locomotor activity (Supplementary Fig. S1a,b). The locomotor activity was also evaluated using rotarod (Supplementary Fig. S1c). There were no significant differences between wild-type (WT) and *Mincle*^−/−^ mice in any of the parameters, based on data obtained from the open-field and rotarod tests. We concluded that *Mincle*^−/−^ mice did not possess considerable dysfunction in locomotor activity and anxiety-like behaviours.

**Figure 1:**
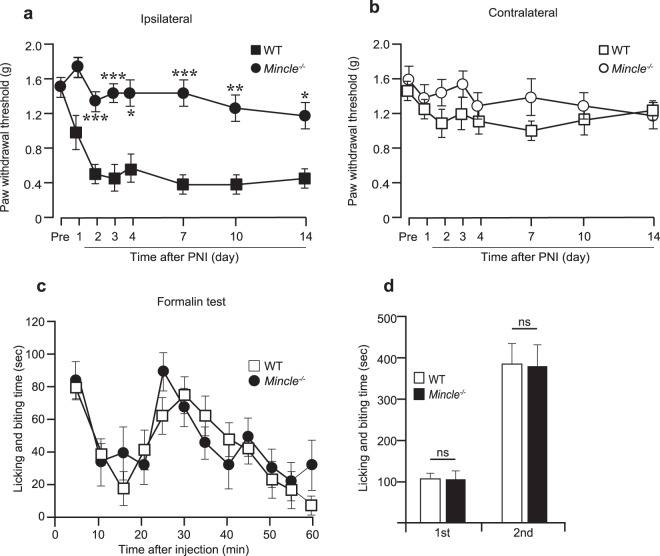
Mincle deficiency attenuates mechanical allodynia after peripheral nerve injury. Fifty percent paw withdrawal threshold (PWT) in ipsi- (**a**) or contra- (**b**) lateral side of WT and *Mincle*^−/−^ mice before (pre) and after PNI (*n* = 7 in each group, repeated ANOVA followed by Bonferroni *post hoc* test, *P < 0.05, **P < 0.01, ***P < 0.001 vs. the ipsilateral side of WT mice). (**c**) The duration (sec) of nociceptive behaviours after formalin injection (*n* = 7 in each group, MANOVA). (**d**) Total durations (sec) of nociceptive behaviours for the first phase (0–10 min) and for the second phase (10–60 min) in the formalin test (*n* = 7 in each group, Student’s t test).

Next, we investigated whether Mincle contributes to the mechanical hypersensitivity after PNI. Basal mechanical sensitivity of *Mincle*^−/−^ mice was not different from that of WT mice (Fig. 1a,b). PNI induced mechanical allodynia in the ipsilateral hind paw of WT mice as previously reported^19^. However, *Mincle*^−/−^ mice exhibited attenuation of mechanical allodynia in the ipsilateral hind paw, and the paw withdrawal thresholds (PWTs) of these mice were significantly higher than those of WT mice (Fig. 1a). The contralateral PWTs after PNI remained unchanged in *Mincle*^−/−^ as well as WT mice (Fig. 1b). We also evaluated the pain behaviour of *Mincle*^−/−^ mice by using the formalin test, a different type of pain model. Pain responses (licking and biting) in both the first and second phases after formalin injection were indistinguishable between WT mice and *Mincle*^−/−^ mice (Fig. 1c,d). These results indicate that Mincle is required for the development of tactile allodynia after PNI but not pain responses induced by the injection of formalin.

### PNI induces expression of *Mincle* mRNA in injured spinal nerves

Next, we tried to identify tissue(s) and/or cell type(s) expressing Mincle after PNI. First, we assumed that Mincle would be expressed in spinal microglia because: (1) it has been well established that spinal microglia are activated and show massive proliferation after PNI, and these changes are important in the pathogenesis of nerve injury-induced pain hypersensitivity^20–24^; (2) in these reports, TLRs expressed in the microglia contribute to neuropathic pain pathology^1,5,6,20^. Then, we tested the expression of *Mincle* mRNA in the spinal dorsal horn of WT mice before and after PNI using real-time PCR. However, *Mincle* mRNA was found to be too low to be detected and remained at undetectable levels even after PNI, unexpectedly. As a positive control of microglial gene expression, mRNA encoding ionized calcium-binding adaptor molecule-1 (Iba1), a microglial marker, was also examined by the same method. The expression of *Iba1* mRNA was detected to be substantial even before PNI and increased after PNI, as expected (Supplementary Fig. S2a). These results indicate that Mincle expression in spinal microglia seems to be largely limited and not to be induced after PNI.

**Figure 2:**
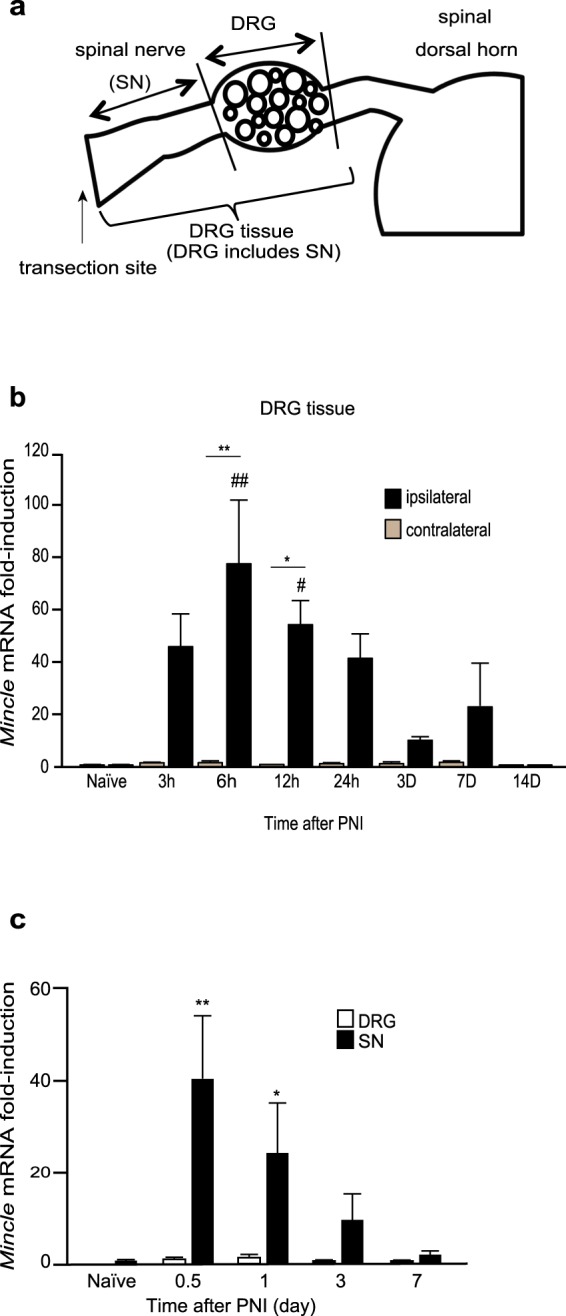
PNI induces expression of *Mincle* mRNA in injured spinal nerve. (**a**) Schematic representation of the tissue used for real-time PCR analysis. (**b,c**) The time course of *Mincle* mRNA expression before (naïve) and after PNI in WT mice. The expression level of *Mincle* gene was normalized to that of *Gapdh* gene. (**b**) Total RNA was obtained from the DRG tissue, which includes the SN (**a**). Data are presented as fold-induction compared to naïve contralateral side (*n* = 4–5 in each group, two-way ANOVA followed by Tukey’s *post hoc* test, ^#^*P* < 0.05, ^##^*P* < 0.01 vs. naïve, *P < 0.05, **P < 0.01 vs. contralateral side). (**c**) Total RNA was obtained from the SN or DRG (**a**). Bar graphs show fold-change relative to the naïve SN (*n* = 3–7 in each group, one-way ANOVA followed by Dunnett *post hoc* test, ^*^P < 0.05, **P < 0.01 vs. naïve controls). Values are mean ± SEM.

Next, we examined *Mincle* mRNA expression in the DRG tissue, as a number of studies have reported that the expression levels of various genes are changed after PNI in the DRG and that the changes are thought to markedly contribute to the generation of mechanical allodynia^3,25–27^. The DRG tissue that we used for the initial experiments included the (injured) SN (Fig. 2a). The expression of *Mincle* mRNA was barely detectable in the naïve mice. However, once the SN was transected, a rapid and marked increase of *Mincle* mRNA was observed in the ipsilateral but not the contralateral DRG tissue (Fig. 2b). To identify the part of the tissue containing *Mincle* mRNA, we then divided the DRG tissue into the DRG itself and SN (Fig. 2a). A remarkable increase in *Mincle* mRNA was observed in the SNs (Fig. 2c) with virtually the same time course as observed in the DRG tissues (Fig. 2b), while only a slight increase was observed in the DRGs. These results indicate that Mincle is expressed in cells in the injured SN, including immune cells, such as the neutrophils, which are known to infiltrate the injured SN after PNI, or the SN resident cells such as the Schwann cells.

### *Mincle* mRNA is expressed in the cells infiltrating the injured nerve

**Figure 3:**
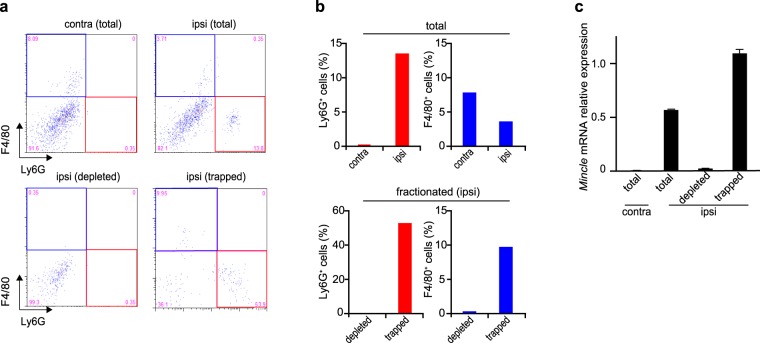
Elimination of *Mincle* mRNA by depletion of neutrophils and macrophages/monocytes from injured SN cells. (**a**) Representative flow cytometry dot plots of Ly6G^+^ and/or F4/80^+^ cells and (**b**) the proportions of these cells in samples of contralateral total, ipsilateral total, ipsilateral depleted fraction, and ipsilateral trapped fraction cells 12 h after PNI. (**a**) Lower-right (red frame) and upper-left (blue frame) quadrant of the panels representing neutrophils (Ly6G^+^) and macrophages/monocytes (F4/80^+^), respectively. Note that neutrophils and macrophages/monocytes were adequately removed from the ipsilateral depleted fraction (lower-left panel in a and lower panels in b). (**c**) The *Mincle* mRNA expression in each sample. The expression level of *Mincle* gene was normalized to that of *Gapdh* gene. PCR amplifications were done in triplicate. Data are representative of two independent experiments. Note that *Mincle* mRNA expression in the ipsilateral depleted fraction was virtually eliminated.

Previous studies have shown that neutrophils are the predominant immune cells migrating to the injured tissue within hours of injury, reaching a peak at 24 h^3,28^. The recruitment and activation of resident macrophages and invasion of monocytes from the peripheral blood follow neutrophil migration^29,30^. Haematoxylin-stained sections from the injured DRG and SN were observed to assess whether infiltrating cells were found in these tissues. Consistent with a previous report^28^, a large number of infiltrating cells were found in the ipsilateral SN 12 h after PNI (Supplementary Fig. S3a). Most of these cells seemed to be neutrophils because they had a lobular nucleus. Because Mincle is reported to be expressed in activated phagocytes and neutrophils^12,14,15^, we assumed that the possible source of induced *Mincle* mRNA expression observed in the injured SN was the neutrophils. To test this assumption, a mixture of Ly6G- (neutrophil marker) and/or F4/80- (monocytes/macrophages marker) positive cells were depleted from SN samples collected 12 h after nerve injury by using the columns designed for depletion of target cells (Fig. 3a,b). The real-time PCR analysis in each sample (contralateral, ipsilateral total, ipsilateral dpleted fraction, and trapped fraction) revealed that *Mincle* mRNA was virtually eliminated in the depleted fraction as expected, while it was preserved in the trapped fraction (Fig. 3c). The result indicated that the major source of *Mincle* mRNA 12 h after PNI was Ly6G- and/or F4/80-positive cells.

**Figure 4:**
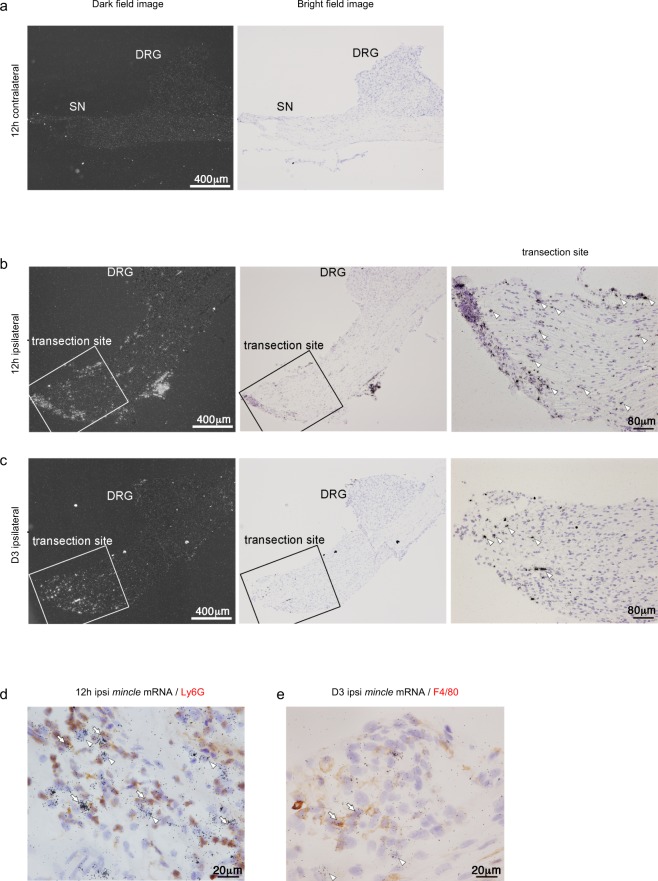
PNI up-regulates *Mincle* mRNA at the transection site. (**a**–**c**) Dark field and bright field images of the same area of ISHH revealed mRNA distribution of *Mincle* mRNA after PNI in the L4 DRG and SN, 12 h after PNI, contralateral (**a**), ipsilateral (**b**), and 3 days after PNI, ipsilateral site (**c**). Arrowheads indicate positive cells. Scale bar: Dark field and bright field images, 400 μm, transection site, 80 μm. Counterstained with haematoxylin. (**d,e**) Bright-field photomicrographs of combined ISHH for *Mincle* mRNA with immunostaining with Ly6G or F4/80 in the ipsilateral SN. Arrowheads indicate cells single-labelled with ISHH (aggregation of grains). Arrows indicate cells double-labelled with ISHH (aggregation of grains) and IHC (brown staining). Counterstained with haematoxylin. Scale bar: 20 μm.

The localization of *Mincle* mRNA in the DRG tissue (including the SN) was further examined using *in situ* hybridization histochemistry (ISHH). A large number of *Mincle* mRNA-expressing cells were observed in the ipsilateral SN, especially at the transection site, but not in the contralateral SN 12 h after PNI (Fig. 4a,b). Three days after PNI, these cells were still observed in the ipsilateral SN (Fig. 4c), although they were decreased in number. Unlike the ipsilateral SN, *Mincle* mRNA-expressing cells were scarcely found in the ipsilateral DRG at any of the time points observed. These results were consistent with our results obtained using real-time PCR. To determine the cell types expressing *Mincle* after PNI, we combined ISHH for *Mincle* mRNA with immunohistochemistry (IHC) using the cell-type-specific markers. Some of the *Mincle* mRNA signals were found to be colocalised with Ly6G (Fig. 4d) or F4/80 (Fig. 4e) 12 h or 3 days after PNI, respectively. Since *Mincle* mRNA-expressing cells were preferentially located in the epineurium (Supplementary Fig. S4), densities of these cells were quantified separately in the epineurium or nerve. The cell density of *Mincle* mRNA-expressing cells in the epineurium was much higher (7.38/10^4^ μm^2^) than that in the nerve (0.47/10^4^ μm^2^) 12 h after PNI. Majority (70.1%) of these in the epineurium and more than half (54.8%) of these in the nerve showed Ly6G-immunoreactivity. In total, 67.4% of these cells were identified as neutrophils (Supplementary Table S1). The cell density of *Mincle* mRNA-expressing cells in the epineurium was still higher (1.46/10^4^ μm^2^) than that in the nerve (0.76/10^4^ μm^2^) 3 days after PNI, however, it was much lower than that 12 h after PNI. Among these cells, 31.4% of these in the epineurium and 45.4% of these in nerve showed F4/80-immunoreactivity. In total, 41.8% of these cells were identified as monocytes/macrophages (Supplementary Table S2). These results indicate that the neutrophils and monocytes/macrophages express *Mincle* mRNA after PNI. In addition, *Mincle* mRNA-expressing cells that showed neither Ly6G nor F4/80 immunoreactivities were also found. Although we did not perform further analysis, these cells may be Schwann cells as judged from their morphology.

### Leucocyte infiltration in injured SN is comparable between WT and *Mincle*^−/−^ mice

**Figure 5:**
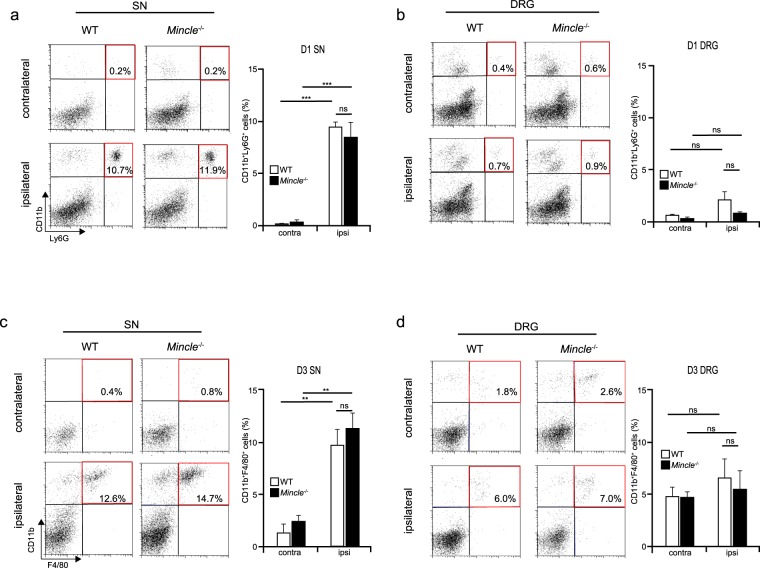
Normal leucocyte infiltration at the injured site of *Mincle*^−/−^ mice. Representative flow cytometry dot plot of CD11b^+^Ly6G^+^ cells in the SN (**a**) and DRG (**b**) 1 day after PNI. Upper-right quadrant (red frame) of the left panels representing infiltrating neutrophils (CD11b^+^Ly6G^+^). The bar graph (right) summarises the proportions of neutrophils in the ipsilateral or contralateral tissues of WT or *Mincle*^−/−^ mice (*n* = 5 in each group, one-way ANOVA followed by Tukey’s *post hoc* test, ***P < 0.001 vs. contralateral side). Representative flow cytometry dot plot of CD11b^+^F4/80^+^ cells in the SN (**c**) and DRG (**d**) 3 days after PNI. Upper-right quadrant (red frame) of the left panels representing infiltrating monocytes/macrophages (CD11b^+^F4/80^+^). The bar graph (right) summarises the proportions of monocytes/macrophages in the ipsilateral or contralateral tissues of WT or *Mincle*^−/−^ mice (*n* = 4 in each group, one-way ANOVA followed by Tukey’s *post hoc* test, **P < 0.01 vs. contralateral side). Values are mean ± SEM.

As described above, Mincle was expressed in leucocytes, which infiltrated the injured SN after PNI. Because Mincle is reported to trigger neutrophil infiltration into the injured tissue^15^, we investigated whether *Mincle*-deficiency would affect migration of immune cells to the injured SN. First, we attempted to analyse the infiltrating cells in a haematoxylin-stained section of the injured DRG tissue (that included the SN) to compare between WT and *Mincle*^−/−^ mice. Although a large number of infiltrating cells were observed in the injured SN (Supplementary Fig. S3a,b), we found it difficult to quantify the number of infiltrating cells by this method, because haematoxylin staining posed limitations with cell identification and it was difficult to obtain sections of uniform size. To solve these problems, we employed the flow cytometry as a rapid and reliable screening method for the characterization and quantification of migrating leucocytes to the injured tissue after PNI. We focused on the neutrophils on day 1 and monocytes/macrophages on day 3 after PNI, based on the observation of these cells in our ISHH experiment. Consistent with previous reports^28–30^, migration of neutrophils (CD11b^+^Ly6G^+^) or monocytes/macrophages (CD11b^+^F4/80^+^) were observed in the injured but not uninjured SN of WT mice 1 day and 3 days after PNI, respectively (Fig. 5a,c). Although the proportions were smaller than in the SN, these cells were also observed to migrate to the ipsilateral but not contralateral DRG (Fig. 5b,d). Unlike in a previous report^15^, infiltrating cells were also observed in the ipsilateral SN and DRG of *Mincle*^−/−^ mice, and the proportions of leucocytes were comparable between WT and *Mincle*^−/−^ mice. These results indicate that Mincle is not involved in leucocyte migration to the injured tissue in this model.

### Mincle affects the induction of neuropathic pain-related genes in the DRG and spinal dorsal horn after PNI

**Figure 6:**
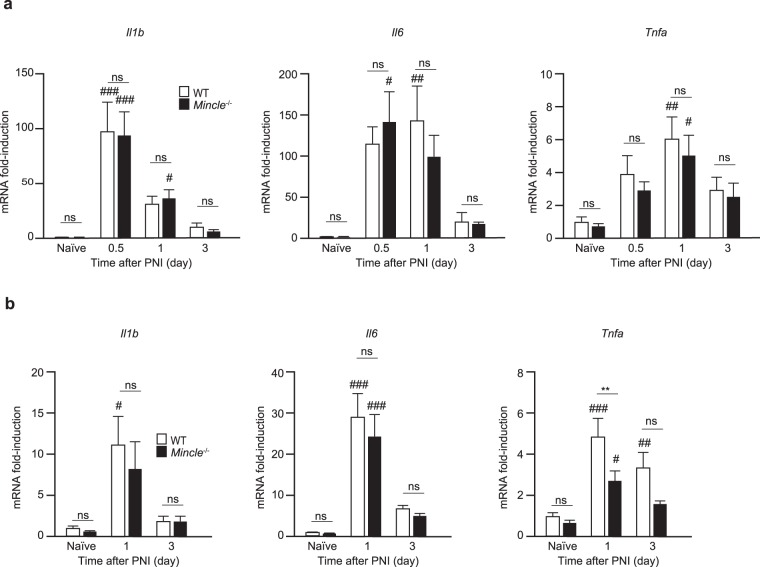
PNI-induced *Tnfa* expression in the injured DRG is reduced in *Mincle*^−/−^ mice. Total RNA was isolated from the SN (**a**) and DRG (**b**) from WT and *Mincle*^−/−^ mice before (naïve) and after PNI. Gene expression was measured using real-time PCR and presented as fold-induction compared to naïve WT mice (*n* = 3–6 in each group, two-way ANOVA followed by Tukey’s *post hoc* test, **P < 0.01 vs. WT mice, ^#^*P* < 0.05, ^##^*P* < 0.01, ^###^*P* < 0.001 vs. naïve controls). Values are mean ± SEM.

Previous reports have shown that inflammatory cytokines (IL-1β, IL-6, and TNF-α) are released from activated immune cells, and are involved in the pathogenesis of neuropathic pain^2,26,27,31^. In the DRG, neurons, satellite cells, and resident macrophages are reported to be activated and to modulate neuronal activity after PNI^3,25^. Therefore, we investigated the temporal expression of these cytokines in the DRG and SN after PNI using real-time PCR. In the SNs, the expression of these cytokines in *Mincle*^−/−^ mice was comparable to that in WT mice (Fig. 6a). In the DRGs, however, induction of *Tnfa* mRNA was significantly suppressed in *Mincle*^−/−^ mice at day 1 after PNI (Fig. 6b). These results suggest that after PNI, reactions induced by Mincle activation in the SN affect the expression of TNF-α in the DRG.

**Figure 7:**
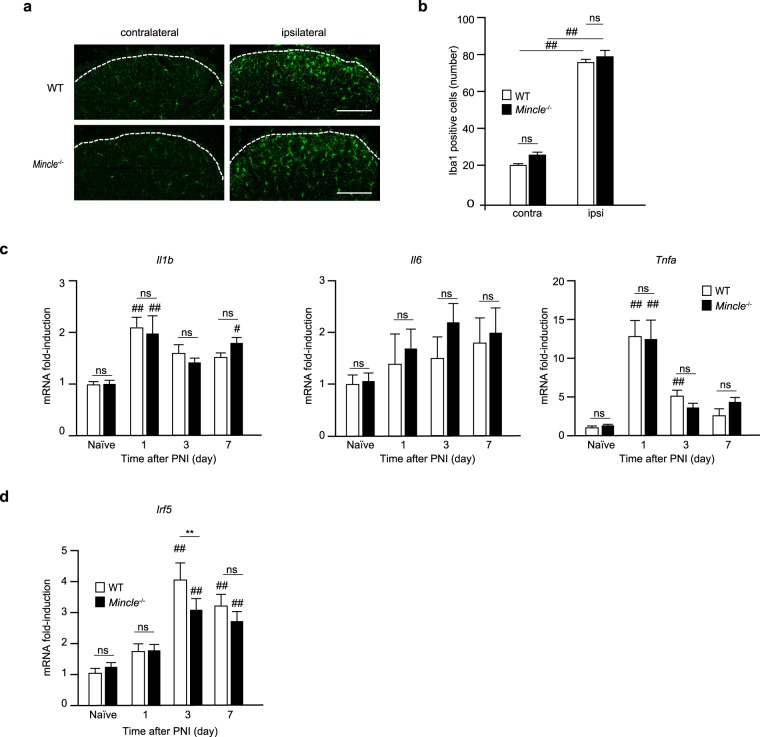
Mincle deficiency inhibits complete activation of spinal microglia after PNI. (**a**) Representative images showing Iba1-immunoreactivity in the L4 spinal dorsal horn of WT and *Mincle*^−/−^ mice 7 days after PNI (Scale bar: 100 μm). (**b**) Number of Iba1-immunoreactive cells counted in the ipsilateral and contralateral dorsal horn of WT and *Mincle*^−/−^ mice (one-way ANOVA followed by Tukey’s *post hoc* test, ^##^*P* < 0.01 vs. contralateral side). Real-time PCR analysis of mRNA of proinflammatory cytokines (**c**) and neuropathic pain-related gene (**d**) in the ipsilateral spinal dorsal horn of WT and *Mincle*^−/−^ mice before (naïve) and after PNI. Bar graphs show fold-change compared to naïve WT mice (*n* = 3–7 in each group, one-way ANOVA followed by Tukey’s *post hoc* test. **P < 0.01 vs. WT mice, ^#^*P* < 0.05, ^##^*P* < 0.01 vs. naïve controls). Values are mean ± SEM.

As described above, the importance of microglial proliferation and activation in neuropathic pain after PNI is well established^20–22^. Because PNI failed to induce mechanical allodynia in *Mincle*^−/−^ mice, we tested the possibility that *Mincle*-deficiency might impair microglial proliferation in the ipsilateral dorsal horn after PNI. As reported previously^21,23^, Iba1-immuno-reactivity revealed massive proliferation of spinal microglia in the ipsilateral dorsal horn of WT mice 7 days after PNI (Fig. 7a). Surprisingly, proliferation of spinal microglia was observed to a similar degree in *Mincle*^−/−^ mice (Fig. 7a,b). This discrepancy between microglial proliferation and reduction of mechanical allodynia has been also described in previous reports of neuropathic pain models in mice lacking P2Y_12_R^22^, interferon regulatory factor-5 (IRF5)^23^, and DNAX-activating protein of 12 kDa (DAP12)^24,32^. Therefore, we assumed that *Mincle*-deficiency might negatively affect complete activation of spinal microglia. To test this idea, we quantified the expression of inflammatory cytokines (*Il1b, Il6*, and *Tnfa*) using real-time PCR, since these cytokines have been reported to be expressed by activated microglia^1,20,23^. Unexpectedly, expression of these inflammatory cytokines in *Mincle*^−/−^ mice was comparable to that in WT mice (Fig. 7c). We also examined the expression of *Irf5*, because this gene was reported to be expressed in microglia and to be involved in neuropathic pain^21,23,33^. Interestingly, real-time PCR analysis revealed that the induction of *Irf5* mRNA expression was significantly suppressed in *Mincle*^−/−^ mice 3 days after PNI (Fig. 7d). These results suggest that after PNI, reactions induced by Mincle activation in the SN affect the expression of IRF5 in the spinal dorsal horn.

### Increase of *Mincle* mRNA in injured SN depends on MyD88 in neuropathic pain model

**Figure 8:**
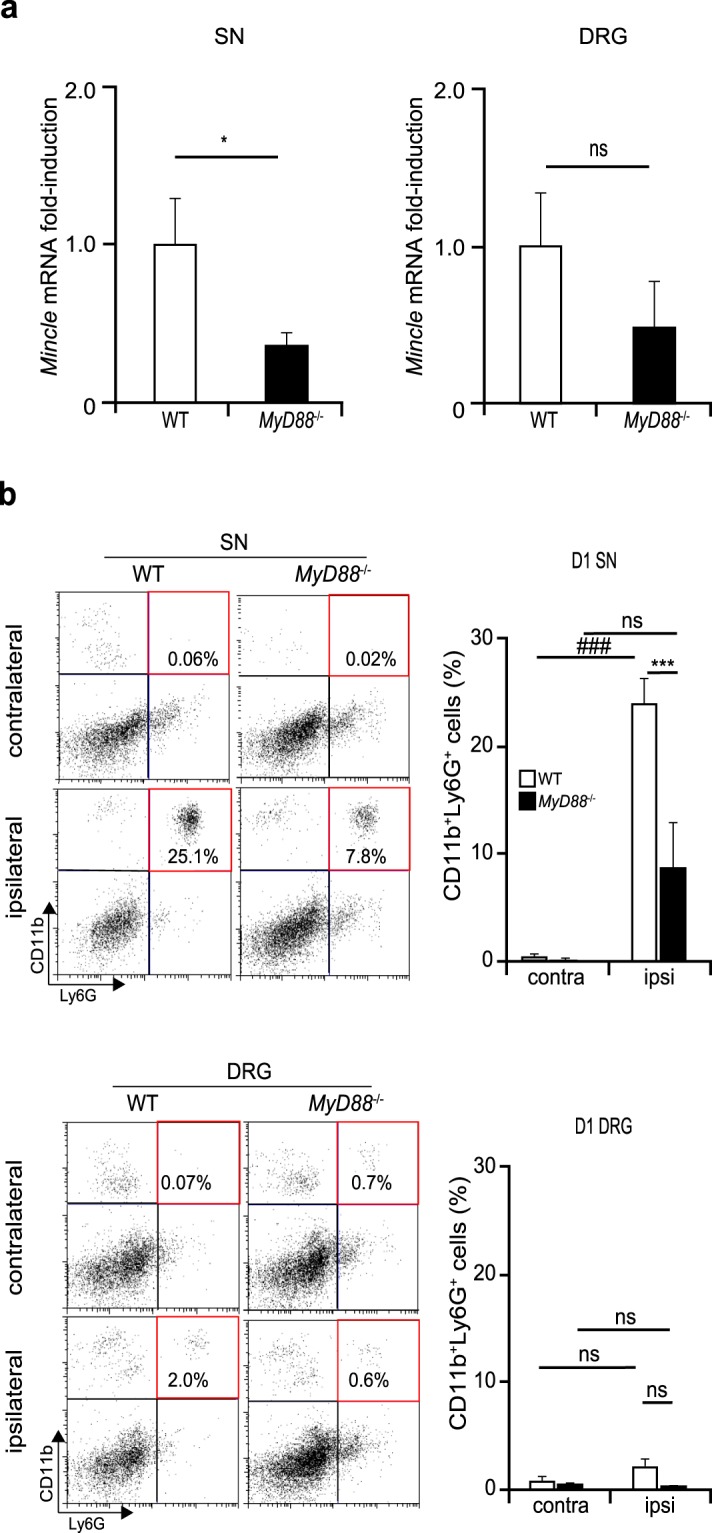
Increase of *Mincle* mRNA in injured SN depends on MyD88 in neuropathic pain model. (**a**) Real-time PCR analysis of *Mincle* mRNA in the injured SN and DRG 12 h after PNI in WT and *MyD88*^−/−^ mice. Bar graphs show fold-change compared to naïve WT mice (*n* = 3 in each group, Student’s t test. *P < 0.05 vs. WT mice). (**b**) Representative flow cytometry dot plot of CD11b^+^Ly6G^+^ cells (left) in the SN (upper) and DRG (lower) 1 day after PNI. Upper-right quadrant (red frame) representing infiltrating neutrophils (CD11b^+^Ly6G^+^). The bar graph (right) summarises the proportions of neutrophils in the ipsilateral or contralateral tissues of WT or *MyD88*^−/−^ mice (*n* = 3 in each group, one-way ANOVA followed by Tukey’s *post hoc* test, ***P < 0.001 vs. WT mice, ^###^*P* < 0.001 vs. contralateral side). Values are mean ± SEM.

It has been reported that the TLR signalling pathways are known to contribute to Mincle expression^12,34^. The MyD88 protein is one of the major adaptor proteins that mediate the TLR signalling pathways^8^, and it is also known to mediate Mincle expression^35^. On the other hand, previous studies have reported that *MyD88*^−/−^ mice showed reduced mechanical allodynia in the neuropathic pain model^36^ and MyD88 in nociceptors contributes to the infiltration of innate and adaptive immune cells into injured DRG after PNI^37,38^. Therefore, we examined whether the increase of *Mincle* mRNA in the injured DRG and SN were cancelled in *MyD88*^−/−^ mice 12 h after PNI. Real-time PCR analysis revealed that the nerve injury-induced increase of *Mincle* mRNA in the injured SN was significantly attenuated in *MyD88*^−/−^ mice compared to that in WT mice (Fig. 8a). Next, we investigated whether *MyD88*-deficiency affects the migration of leucocytes into the SN and DRG. Flow cytometry analysis revealed that the proportion of neutrophils (CD11b^+^Ly6G^+^) in the injured SN 1 day after PNI was significantly decreased in *MyD88*^−/−^ mice compared to that in WT mice (Fig. 8b). Based on these results, it is suggested that the migration of neutrophils appears to be MyD88-dependent in this model, and that the reduction of *Mincle* mRNA in the SN of *MyD88*^−/−^ mice might be due, at least in part, to the reduced infiltration of neutrophils.

## Discussion

The present study provides the first evidence of the contribution of Mincle to pathological changes in a mouse model of neuropathic pain. The major findings of this study are as follows: (1) mechanical allodynia induced by PNI was attenuated in *Mincle*^−/−^ mice; (2) *Mincle* mRNA was markedly induced in the injured SN, and migrating immune cells, especially neutrophils, were considered to be the major source of *Mincle* mRNA; (3) *Mincle*-deficiency affected the expression of certain genes, which are reported to contribute to neuropathic pain after PNI.

Recent studies on neuropathic pain have revealed that the TLRs, a family of PRRs, are involved in neuropathic pain after PNI^6,7^. It has been reported that when a peripheral nerve is injured, a variety of immune cells are recruited to the site of injury and release numerous cytokines and chemokines, which coordinate the cellular response to nerve injury and directly alter the sensory transduction properties and ongoing activity of primary afferents^1,3,4^.

These immune molecules, especially the proinflammatory cytokines, are also known to be upregulated in the DRG and spinal dorsal horn^3,26,27^. While a significant number of studies support a link between this disease and the TLRs, the current data demonstrate that Mincle also plays a role in triggering such sequential reactions.

Because PNI failed to induce mechanical allodynia in *Mincle*^−/−^ mice, we compared the expression profile of cytokines and pain-related molecules in the DRG and spinal dorsal horn between *Mincle*^−/−^ and WT mice before and after PNI. Induction of *Tnfa* in the DRG and *Irf5* in the spinal dorsal horn were found to be significantly suppressed in *Mincle*^−/−^ mice compared to those in WT controls. This result suggested that Mincle-mediated signals (and the subsequent reactions) in the injured SN play a role in the upregulation of *Tnfa* and *Irf5* mRNA in the DRG and spinal dorsal horn, respectively. A growing body of literature demonstrates that TNF-α plays an important role in initiating inflammatory reactions of the innate immune processes and stimulation of inflammatory cells in injured nerves, and contributes to the generation of pain^27,31,39,40^. The direct application of TNF-α is known to induce ectopic firing in Aδ-, Aβ-, and C-fibers^39,40^, and also induce thermal hyperalgesia and mechanical allodynia^41–43^. TNF-α is also reported to enhance the sensitivity of nociceptors through the phosphorylation of Nav1.8 and Nav1.9 sodium channels^44^. Furthermore, a previous study has shown that TNF-α can increase excitatory synaptic transmission directly at the central terminal of primary afferents, and glutamatergic currents in the dorsal horn neurons^1,45^. After nerve injury, TNF-α is synthesized and probably released by various cell types in the DRG, such as the satellite glial cells, and even in sensory neurons^46,47^. Ogawa *et al*. demonstrated that silencing of TNF-α in the injured DRG reduces mechanical allodynia after PNI^46^. Together with these studies, our results indicate that TNF-α produced in the DRG by Mincle-dependent mechanisms might play an important role in the pathogenesis of neuropathic pain. It is not clear whether TNF-α produced in DRG neurons can be released from the central terminals of primary afferents, while some of the cytokines and chemokines such as CCL2, CCL21, and colony-stimulating factor1 (CSF1) are known to be released^1,2,21,24^.

In the spinal dorsal horn where the central axons of DRG neurons terminate, microglia have been reported to proliferate, exhibit marked changes in cell surface protein expression, and release various chemical mediators after PNI^4,20^. These responses are reported to be involved in the pathology of neuropathic pain. Because mechanical allodynia was ameliorated in *Mincle*^−/−^ mice after PNI, we expected that the proliferation of spinal microglia would be attenuated. However, the number of these cells in *Mincle*^−/−^ mice was found to be comparable to that in WT mice after PNI. This discrepancy between microglial proliferation and the ameliorated mechanical allodynia has been also reported in mice lacking P2Y_12_R^22^, IRF5^23^, and DAP12^24,32^. Recently, CSF1, which is synthesized in DRG neurons and released from their central terminals to the spinal dorsal horn after PNI, has been reported to promote the proliferation of spinal microglia^24^. Consistent with this, *Csf1* mRNA was found to be also induced in the DRG of *Mincle*^−/−^ mice (Supplementary Fig. S2b). Together with other studies, the discrepancy mentioned here shows that induction of mechanical allodynia after PNI required not only microglial proliferation but also change(s) in microglial profile(s). IRF5 expressed in spinal microglia is reported to contribute to neuropathic pain after PNI^23^. In the current experiments, we found that upregulation of this *Irf5* in the spinal dorsal horn after PNI was significantly suppressed in *Mincle*^−/−^ mice. This result is consistent with our data from behavioural experiments. Although IRF5 is reported to drive P2X4R^+^-reactive microglia after PNI^23^, we found the expression of *P2rx4* in *Mincle*^−/−^ mice to be similar to that in WT mice (data not shown). A previous study also reported that IRF5 is critical for expression of M1 macrophage-related genes including proinflammatory cytokines (*Il1b, Il6*, and *Tnfa*)^48^; however, no differences were detected in the expression of these genes between *Mincle*^−/−^ and WT mice in our experiments. Unidentified downstream pathways from IRF5 might also be involved in the induction of neuropathic pain after PNI.

After PNI, *Mincle* mRNA was induced only in the ipsilateral DRG tissue that included the SN, but not in the spinal dorsal horn. Since previous studies have shown that TLRs are expressed in the microglia^5–7^ and recognize DAMPs as their agonists directly^6^, we expected that Mincle would be also expressed in spinal microglia after PNI. However, *Mincle* mRNA in the ipsilateral dorsal horn remained at undetectable levels even after PNI while microglial proliferation (Fig. 7a,b) and upregulation of *Iba1* mRNA (microglial marker) were observed (supplementary Fig. S2a). Thus, Mincle expression in spinal microglia appeared to be largely limited and was not induced after PNI. In rodent models of cerebrovascular disorders (CVD), Mincle is reported to be expressed in microglia-like cells, neurons, and endothelial cells^17,18^. In the CVD model, the blood-brain-barrier is broken down and blood cells gain access into the cerebral parenchyma. In contrast, no infiltrating microglia-like cells are observed in the PNI model^49^. Thus, *Mincle*-expressing microglia-like cells in the CVD model might be from blood and this could be a reason for the undetectable levels of *Mincle* mRNA in the spinal dorsal horn even after PNI in our model.

In the DRG tissue containing the SN, we further dissected the region of *Mincle* mRNA expression, and remarkable *Mincle* mRNA induction was observed in the injured SN, but not in the DRG. These results indicate a possibility that the immune cells that migrated to the injured SN may be the source of *Mincle* mRNA. This possibility was strengthen by the results of depletion experiments in which *Mincle* mRNA was virtually eliminated after depletion of neutrophils and macrophages/monocytes from the cells in injured SN. The presence of *Mincle* mRNA-expressing cells in injured SN was also confirmed by ISHH. Furthermore, ISHH with IHC also revealed the *Mincle* mRNA-expressing cells 12 h after PNI were mostly the migrating neutrophils, while those 3 days after PNI were monocytes/macrophages and some other unidentified cells. However, none of DRG neurons, including nociceptors, were found to express *Mincle* mRNA. Since systemic depletion of the neutrophils or macrophages reduce mechanical hypersensitivity after PNI^28,50^, it is suggested that these infiltrating cells expressing Mincle are required to generate neuropathic pain.

In some cases, *Mincle* mRNA signals were also detected in a population of cells that were neither neutrophils nor monocytes/macrophages. These cells may be other types of migrating immune cells or resident cells in the SN such as the Schwann cells. It has been reported that Schwann cells also proliferate and release inflammatory mediators after PNI and could play a key role in the development of neuropathic pain states^4,51^. Taken together, current results suggest that the *Mincle*-expressing cells for induction of neuropathic pain likely include neutrophils and macrophages, however, it would be possible that some unidentified cell types, being assumed to include Schwann cells, may also contribute to neuropathic pain.

When a peripheral nerve is injured, various immune cells are recruited to the site of injury. Mincle is known to promote neutrophil infiltration into the necrotic thymus tissue after whole body irradiation^15^. Thus, we assumed that migration of leucocytes to the injured nerve would be reduced in *Mincle*^−/−^ mice. However, the migration of leucocytes into the injured nerve was comparable between WT and *Mincle*^−/−^ mice. In addition, mRNA expression of the *Ccl2* chemokine, which is known to regulate the migration of leucocytes, also showed similar induction in the injured SN and DRG between WT and *Mincle*^−/−^ mice after PNI (Supplementary Fig. S2c). The TLRs are also known to be required for the induction of leucocyte migration into the injured nerve^51^. Numerous studies have shown the TLRs and their signalling protein MyD88 are involved in neuropathic pain and deletion of these genes has resulted in reduced tactile allodynia in the rodent model of neuropathic pain^6,36–38^. The following mechanisms have been proposed: (1) *MyD88*-deficiency results in less DRG reactivity to nerve injury^36^ and (2) MyD88 is required for microglial proliferation in the ipsilateral dorsal horn^36^. In the experiments using *MyD88* conditional knockout mice, neuronal MyD88 is reported to be required for peripheral nerve injury-induced CCL2 up-regulation and macrophage infiltration into the DRG, and microglial proliferation^37^. In addition, MyD88 in nociceptive neurons is reported to be necessary for driving innate and adaptive immunity in DRGs^38^. Consistent with these observations, infiltration of neutrophils and monocytes/macrophages in the injured SN of *MyD88*^−/−^ mice were impaired in our experiments. In these *MyD88*^−/−^ mice, impairment of the increased *Mincle* mRNA in injured SN was also observed. These results strengthen the idea that the main source of *Mincle* mRNA in the injured SN was the migrating neutrophils and monocytes/macrophages. Alternatively, the impaired increase of *Mincle* mRNA in *MyD88*^−/−^ mice may be due to the lack of TLR signalling because TLR ligands, such as lipopolysaccharides are known to induce *Mincle* mRNA expression^12,52^. Taken together with our results, it was suggested that Mincle may be involved at least in part in the MyD88-dependent mechanical allodynia observed in previous studies.

In this study, we demonstrated the contribution of Mincle to neuropathic pain pathology. *Mincle* mRNA was induced in migrating immune cells and unidentified cells in the injured SN. Recently, β-glucosylceramide (GlcCer), a glycolipid, was reported as a newly identified endogenous ligand for Mincle^16^. Glycolipids including β-GlcCer have been reported to be abundant in the nervous system including the brain^53,54^. Thus, β-GlcCer may be a possible candidate endogenous ligand recognized by Mincle in the neuropathic pain model. After PNI, β-GlcCer may be released or exposed in the injured region within the SN as DAMPs and *Mincle*-expressing cells may recognize it and activate subsequent reactions in the DRG and spinal dorsal horn, leading to neuropathic pain. It has been reported that TNF-α can induce *Irf5* mRNA in primary cultures of healthy human epidermal keratinocytes^55^. Together with our result, it is conceivable that TNF-α induced in the DRG by Mincle dependent reaction(s) in the SN would be released in the spinal dorsal horn and induce IRF5 expression in the microglia which is known to result in neuropathic pain. Although further investigation is required, our results showed that Mincle may be involved in the pathology of neuropathic pain, and blockade of Mincle dependent pathways may provide new therapeutic strategies for neuropathic pain.

## Methods

### Animals

All experiments were approved by the Saga University Animal Care and Ethical Use Committee (#27-064-0, #28-063-0, and #28-027-0) and animals were treated in accordance with the Fundamental Guidelines for Proper Conduct of Animal Experiments and Related Activities in Academic Research Institutions’ (Ministry of Education, Culture, Sports, Science and Technology of Japan) and the ethical guidelines of Saga University. All animal experiments were performed in male mice aged from 10 to 20 weeks at the initiation of the experiments.

*Mincle*-deficient (*Mincle*^−/−^) mice that had been backcrossed to C57BL/6 mice for at least eight generations^14^ were used, and C57BL/6 (SLC, Japan) were used as the wild type (WT) controls. MyD88-deficient (*MyD88*^−/−^) mice that had been backcrossed to C57BL/6 were also used.

Mice were housed in groups of 1-5 per cage at a temperature of 23.0 ± 2 °C and humidity of 55 ± 5% with a 12-h light-dark cycle and were fed food and water *ad libitum*.

### Neuropathic pain model

We used the spinal nerve injury model^56^ with some modifications^19^. Under isoflurane (2%) anaesthesia, a small incision was made at L3-S1, and the paraspinal muscle and fat were removed from the L5 transverse process. The part of the transverse process was removed, and then the left L4 spinal nerve was carefully isolated and cut. The muscles and skin were closed in two layers with a 6–0 silk suture.

### Assessment of mechanical sensitivity

To assess mechanical sensitivity, mice were placed individually in a transparent glass cylinder and separated from each other by an opaque divider, which was placed on a wire mesh, and habituated to the test apparatus for 1 h prior to data collection. After that, calibrated von Frey filaments (0.02~2.0 g, Stoelting, Wood Dale, IL) were applied to the mid plantar surface of the hind paws of mice with or without PNI, until they withdrew their paws in response to the mechanical stimuli. The 50% paw withdrawal threshold was determined using the up-down method^57^.

### Formalin test

Mice were placed in individual boxes and allowed to habituate to the new environment for 15 min. Subsequently, formalin (5%, 20 μL) was subcutaneously injected into the dorsum of the hind paw. The duration of licking and biting responses to the injected hind paw was recorded as a nociceptive response, for 60 min at 5 min intervals after the injection^19^. An initial acute phase [first phase, during the first 10 min after the formalin injection] was followed by a relatively short quiescent period and then by a prolonged tonic response [second phase, beginning 10 min after the formalin injection]. The first phase is presumed to be a result of a direct activation of the primary afferent nociceptors, and the second phase involves peripheral inflammatory events and central sensitization^58^. The cumulative time of nociceptive behaviours during the first and second phases were analysed separately.

### Real-time PCR

Mice were deeply anaesthetized with sodium pentobarbital (50 mg/kg) and then sacrificed by transcardial perfusion with 20 mL of RNA-later (Thermo Fisher Scientific, Waltham, MA) and tissues were removed immediately. RNA extraction was carried out using RNeasy Lipid Tissue Mini Kit (Qiagen, Valencia, CA) according to the manufacturer’s instructions. After removal of the genomic DNA by treatment with DNase (Wako Nippon Gene, Osaka, Japan), complementary DNA synthesis was performed using the ReverTra Ace qPCR RT Master Mix (Toyobo, Osaka, Japan). Real-time PCR was performed using the Thunderbird SYBR qPCR Mix (Toyobo) with a StepOnePlus (Applied Biosystems, Foster City, CA) and the data were analysed using StepOne Software version 2.3 (Applied Biosystems). PCR amplifications were performed at 95 °C for 60 s, and 95 °C for 5 s, 60 °C for 30 s for 40 cycles. Ct values for genes of interest were normalized to correspond to those for a reference gene encoding glyceraldehyde-3-phosphate dehydrogenase (Gapdh) and represented as a fold-change to a control, which was calculated using the 2^−ΔΔCt^ method^59^. The sequences of the primers used in this study are presented below.

*Gapdh* (GU214026.1): 5′-AACTTTGGCATTGTGGAAGG-3′ (forward), 5′-ACACATTGGGGGTAGGAACA-3′ (reverse); *Mincle* (NM_019948.2): 5′-CAGTGGCAATGGGTGGATGATAC-3′ (forward), 5′-AGTCCCTTATGGTGGCACAGTC-3′ (reverse); *Il1b* (NM_008361): 5′-AGATGAAGGGCTGCTTCCAAAC-3′ (forward), 5′-GTTGATGTGCTGCTGCGACAT-3′ (reverse); *Tnfa* (NM_013693.3): 5′-AAGCCTGTAGCCCACGTCGTA-3′ (forward), 5′-GGCACCACTAGTTGGTTGTCTTTG-3′ (reverse); *Il6* (NM_031168.1): 5′-CCACTTCACAAGTCGGAGGCTTA-3′ (forward), 5′-GCAAGTGCATCATCGTTGTTCATAC-3′ (reverse); *Irf5* (NM_001252382.1): 5′-TGGGGACAACACCATCTTCA-3′ (forward), 5′-CTGGAAGTCACGGCTTTTGT-3′ (reverse).

### Immunohistochemistry

Mice were deeply anaesthetized with sodium pentobarbital (50 mg/kg) and then sacrificed by transcardial perfusion with 20 mL of 4% paraformaldehyde in 0.1 M sodium phosphate buffer (PB; pH 7.4) before or 7 days after PNI. The L4 segment of the spinal cord was removed, post-fixed in the same fixative at 4 °C overnight and placed in 30% sucrose solution for 24 h at 4 °C. Transverse L4 spinal cord sections (30 μm) were cut, rinsed three times for 10 min in PBS and preincubated with 1% normal donkey serum containing 1% bovine serum albumin and 0.1% triton X-100 in PBS for 30 min. Sections were then incubated overnight at 4 °C with the primary antibody. Rabbit anti-Iba1 (ionized calcium-binding adapter molecule-1, 1:300, Wako Pure Chemical Industries, Osaka, Japan, RRID: AB_839504) was used. After rinsing three times for 10 min in PBS, these sections were incubated with the secondary antibody. Donkey anti-rabbit IgG conjugated Alexa Fluor 488 (1:400, Thermo Fisher Scientific, RRID: AB_141708) was used. TO-PRO-3 (1:200, Molecular Probes, Eugene, OR) was also used for nuclear staining together with the secondary antibody. Three to five sections of the L4 spinal cord segments of each mouse were randomly selected and analysed using a LSM5 Exciter-Zen 2009 (Carl Zeiss, Oberkochen, Germany). Iba1-immunopositive profiles in the dorsal horn were counted if the profile contained TO-PRO-3 positive nucleus.

### Flow cytometry

Mice were deeply anaesthetized using sodium pentobarbital (50 mg/kg) and then sacrificed by transcardial perfusion with 20 mL of cold PBS at 1 day or 3 days after PNI. The L4 DRG and spinal nerve, ipsilateral and contralateral to PNI, were removed immediately and placed in ice-cold PBS. Tissues were treated with collagenase D (1 mg/mL in RPRI 1640 containing 2% FCS, Roche, Mannheim, Germany) for 1 h at 37 °C with gently shaking. The tissues were homogenized by passing through a mesh. Cells were washed with RPRI 1640 (2% FCS) 2 times. The cells were immunostained with rat anti mouse CD11b-APC (0.4 μg/mL, clone: M1/70, Tonbo Biosciences, San Diego, CA, RRID: AB_2621556), rat anti mouse Ly6G-PE (2 μg/mL, clone: 1A8, BD Biosciences, Franklin Lakes, NJ, RRID: AB_394208), and rat anti mouse F4/80-PE (2 μg/mL, clone: BM8.1, Tonbo Biosciences, RRID: AB_2621795). Neutrophils were defined as CD11b^+^ and Ly6G^+^, while monocytes/macrophages were CD11b^+^ and F4/80^+^. The total number of neutrophils and monocytes/macrophages was analysed using a FACS Calibur (BD Biosciences) and FlowJo software (BD Biosciences).

### Depletion of Ly6G^+^ and/or F4/80^+^ cells from injured SNs

The injured and uninjured L4 spinal nerves were removed at 12 h after PNI as described above. To obtain a sufficient number of cells, a sample was collected from 3 animals, and treated together with collagenase D (1 mg/mL in RPRI 1640 containing 2% FCS, Roche) for 1 h at 37 °C with gently shaking. The tissues were homogenized by passing through a mesh. Cells were washed with RPRI 1640 (2% FCS) 2 times. Two-thirds of the cells obtained from injured SNs were incubated with rat anti mouse Ly6G-FITC (2 μg/mL, clone: 1A8, Tonbo Biosciences, RRID_AB_2621704) and rat anti mouse F4/80-PE (2 μg/mL, clone: BM8.1, Tonbo Biosciences) for 30 min on ice, washed with MACS Running buffer (Miltenyi Biotec, Bergisch Gladbach, Germany), and then incubated with anti-FITC and anti-PE antibody-coated immunomagnetic microbeads (Miltenyi Biotec, RRID: AB_244371, AB_244373, respectively) for 15 min on ice. After cells were washed with MACS Running buffer, unlabeled cells were collected (depleted fraction) from the total cells by using LD Columns (Miltenyi Biotec) which were designed for stringent depletion of labelled cells. Cells remaining in the columns (trapped fraction) were also collected. Four SN samples (contralateral total, ipsilateral total, ipsilateral depleted fraction, and ipsilateral trapped fraction) were further analysed. After confirming successful depletion by flow cytometry, RNA extraction from each sample was carried out using RNeasy Micro Kit (Qiagen), and the expression of *Mincle* mRNA was evaluated by real-time PCR in triplicate.

### *In situ* hybridization

Mice were deeply anaesthetized using sodium pentobarbital (50 mg/kg) and then sacrificed by transcardial perfusion with 20 mL of 4% paraformaldehyde in 0.1 M PB 12 h or 3 days after PNI. The L4 whole DRGs were removed, post-fixed in the same fixative at room temperature for 3 h, and the samples were placed in 20% sucrose solution at 4 °C overnight. Then, the samples were rapidly frozen with powdered dry ice, and cut on a cryostat (HM-500-O, Thermo Fisher Scientific) at 8 μm thickness. The sections were mounted onto MAS-coated glass slides (Matsunami, Osaka, Japan). Using the enzyme-digested clones, 35 S UTP-labelled mouse Mincle antisense and sense cRNA probes (cDNA nucleotides 142–673, accession number NM_019948.2; in pCRII TOPO vector) were synthesized. The protocol for ISHH has been described in detail previously^60^. In order to examine the distribution of *Mincle* mRNA, we combined IHC with ISHH. The following antibodies were used: rat anti mouse F4/80 monoclonal antibody (1:1,000, Clone Cl: A3-1, AbD Serotec, RRID:AB_323806), rat anti mouse Ly6G monoclonal antibody (1:1,000, Clone RB6-8C5, AbD Serotec, RRID:AB_2137489). The treatment of sections and methods of double labelling with IHC and ISHH have been described in a previous paper^60^.

### Statistical analysis

Data are represented as mean ± SEM. Statistical significances were determined using repeated analyses of variance (ANOVA) followed by Bonferroni *post hoc* test (Fig. 1a,b), multivariate analysis of variance (MANOVA) (Fig. 1c), two-way ANOVA followed by Tukey’s *post hoc* test (Figs 2b, 6a,b and 7c,d), one-way ANOVA followed by Dunnett’s *post hoc test* (Fig. 2c), Tukey’s *post hoc* test (Figs 5a–d, 7b and 8b), or unpaired Student’s t test (Figs 1d, 8a), using the JMP^®^ Pro 12 software (SAS Institute Inc., Cary, NC). Differences were considered to be statistically significant at *P* < 0.05.

## Supplementary information

Supplementary Information

## Data Availability

The datasets generated during and/or analysed during the current study are available with the corresponding author, and can be accessed on reasonable request.
