# Supplementary material for: Essential roles of C-type lectin Mincle in induction of neuropathic pain in mice
Source: Sci Rep. 2019 Jan 29;9:872. doi: 10.1038/s41598-018-37318-8 (PMC6351622; doi:10.1038/s41598-018-37318-8)
Supplement: Supplementary file 1 — Supplementary Information [file 41598_2018_37318_MOESM1_ESM.pdf]

Supplementary information

Essential roles of C-type lectin Mincle in induction of neuropathic pain in mice

Asako Ishikawa[1], Yasunobu Miyake[2], Kimiko Kobayashi[3], Yuzo Murata[4], Sayaka Iizasa[5], Ei'ichi Iizasa[6], Sho Yamasaki[7], Naomi Hirakawa[1,8], Hiromitsu Hara[6], Hiroki Yoshida[2], Toshiharu Yasaka*[6]

[1]*Department of Anesthesiology & Critical Care Medicine, Faculty of Medicine, Saga University, 5-1-1 Nabeshima, Saga 849-8501, Japan*
[2]*Division of Molecular and Cellular Immunoscience, Department of Biomolecular Sciences, Faculty of Medicine, Saga University, 5-1-1 Nabeshima, Saga 849-8501, Japan*
[3]*Department of Anatomy and Neuroscience, Hyogo College of Medicine, 1-1 Mukogawa-cho, Nishinomiya, Hyogo 663-8501, Japan*
[4]*Division of Histology and Neuroanatomy, Department of Anatomy & Physiology, Faculty of Medicine, Saga University, 5-1-1 Nabeshima, Saga 849-8501, Japan*
[5]*Department of Biological Science and Technology, The United Graduate School of Agricultural Science, Kagoshima University, 1-21-24 Korimoto, Kagoshima 890-8580, Japan*
[6]*Department of Immunology, Graduate School of Medical and Dental Sciences, Kagoshima University, 8-35-1 Sakuragaoka, Kagoshima 890-8544, Japan*
[7]*Division of Molecular Immunology, Medical Institute of Bioregulation, Kyushu University, 3-1-1 Maidashi, Higashi-ku, Fukuoka 812-8582, Japan*
[8]*Pain clinic & Palliative Care Medicine, Saga University Hospital, 5-1-1 Nabeshima, Saga 849-8501, Japan*

*Correspondence should be addressed to T.Y. (email: yasaka@m.kufm.kagoshima-u.ac.jp)

Supplementary information includes the following:
Supplementary Methods
Supplementary References
Supplementary Figures S1-S3

Supplementary Methods

Assessment of motor function and anxiety-like behaviour. The open field (OF) test was used to exclude the possible influence of abnormalities in motor function and/or anxiety on pain-related behaviour because these factors are known to affect to the assessment of pain-related behaviour. The OF apparatus consisted of a grey circular field with grey walls (50 cm in diameter and 40 cm in height) made of vinyl chloride (Muromachi Kikai, Tokyo, Japan) was used and the field was brightly illuminated by overhead fluorescent lightning (500 lx). The floor of the apparatus was concentrically divided into central, inner, and outer areas. Ten-week-old mice (10 animals/group) were individually placed in the centre of the OF without habituation, and their behaviours were observed for 5 min using a video camera equipped with a computational tracking system (Any-maze; Stoelting Co., Wood Dale, IL). We analysed locomotor activity and anxiety-like behaviour, expressed as total distance travelled and time spent in the centre area, respectively[1,2]. We also used a rotarod apparatus (MK-630, Muromachi Kikai, Tokyo, Japan) to assess motor coordination and balance, in accordance with the manufacturer's protocol. One hour prior to data collection, mice were placed on the static cylinder for 5 min for habituation, following which the preliminary trial was conducted, in which they were placed on the rotating cylinder (5 rpm) for 5 min. Mice were then placed on the constant-speed rotarod (12 rpm) for a maximum of 300 s, and the latency to falling from the rotarod was measured.

Visualization of leucocytes within injured spinal nerves (SNs) (Haematoxylin staining). We utilized the immunohistochemistry procedures described in the main text to prepare sections for haematoxylin staining. Twelve hours after peripheral nerve injury (PNI), the L4 SNs were removed and post-fixed in the same fixative at 4°C overnight, following which they were placed in 30% sucrose solution for 24 h at 4°C. Longitudinal L4 SN sections were cut to a thickness of 20 µm using a cryostat (HM-500-O, Thermo Fisher Scientific, Waltham, MA). Sections were stained with haematoxylin for examination using a light microscope (LABOPHOT, Nikon, Tokyo, Japan).

Real-time PCR. We used the same procedure as described in the main text. Expression levels for each gene were normalized to the values for the mouse glyceraldehyde-3-phosphate dehydrogenase (*Gapdh*) gene. The following primer

sequences were used: *Ccl2* (NM_011333.3): 5′ -TCAGCCAGATGCAGTTAACG- 3′ (forward), 5′ -GATCCTCTTGTAGCTCTCCAGC- 3′ (reverse); *Iba1* (D86382.1): 5′ -GATTTGCAGGGAGGAAAAGCT- 3′ (forward), 5′ -AACCCCAAGTTTCTCCAGCAT -3′ (reverse); *Csf1* (BC025593.1): 5′ -TGCTAAGTGCTCTAGCCGAG- 3′ (forward), 5′ -CCCCCAACAGTCAGCAAGAC- 3′ (reverse).

Statistical Analysis. Data are represented as the mean ± SEM. Statistical significance was determined using unpaired Student's t tests or two-way analyses of variance (ANOVA), followed by Tukey's *post hoc* test. The level of statistical significance was set at $P < 0.05$.

Quantification of *Mincle* mRNA expressing cells and their colocalisation with Ly6G- or F4/80-immunoreactivities. To measure positive cell profiles, 12 to 15 sections from the L5 spinal nerve were randomly selected. Double ISH-IHC slides were digitized with a Nikon ECLIPSE 80i microscope connected to a Nikon DXM-1200F digital camera. An image (820.5 x 656.4 μm) of transection site was captured under a 10 x objective. The area of epineurium or nerve was traced (Supplemental Fig. 4) and measured by Fiji software (ImageJ 2.0.0-rc-68 / 1.52g ; Java1.8.0) and all of the positively stained cells in the each area were counted using cell counter plugin.

Supplementary References

1 Archer, J. Tests for emotionality in rats and mice: a review. *Anim. Behav.* **21**, 205-235 (1973).

2 Tamada, K. *et al.* Decreased exploratory activity in a mouse model of 15q duplication syndrome; implications for disturbance of serotonin signaling. *PloS One* **5**, e15126 (2010).

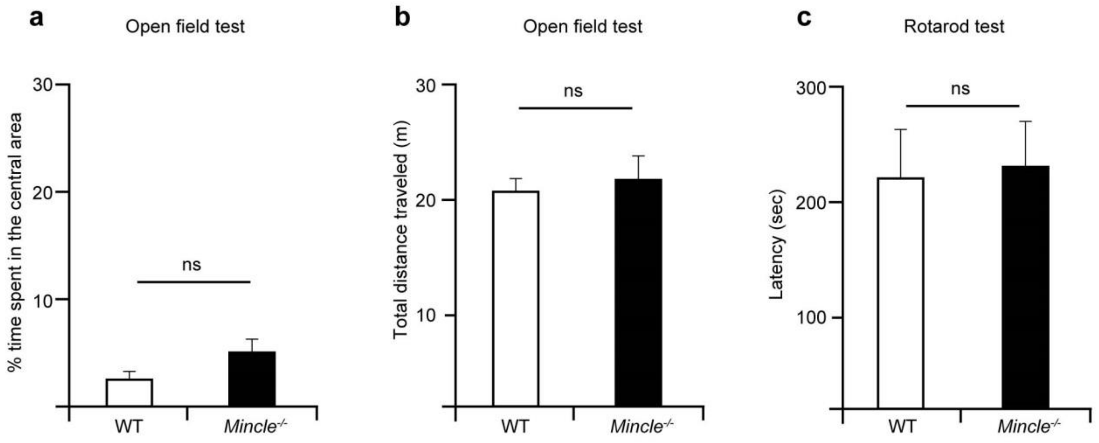

Supplementary Figure 1. *Mincle*$^{-/-}$ mice exhibit normal locomotor activity and anxiety-like behavior. The percentage of time spent in the central area (a) and the total distance travelled (b) during the 5-min testing period of the open field test (n = 10 in each group, Student's t test). (c) The latency to fall from the rotarod (n = 10 in each group, Student's t test). Values are represented as the mean ± SEM. WT: wild-type.

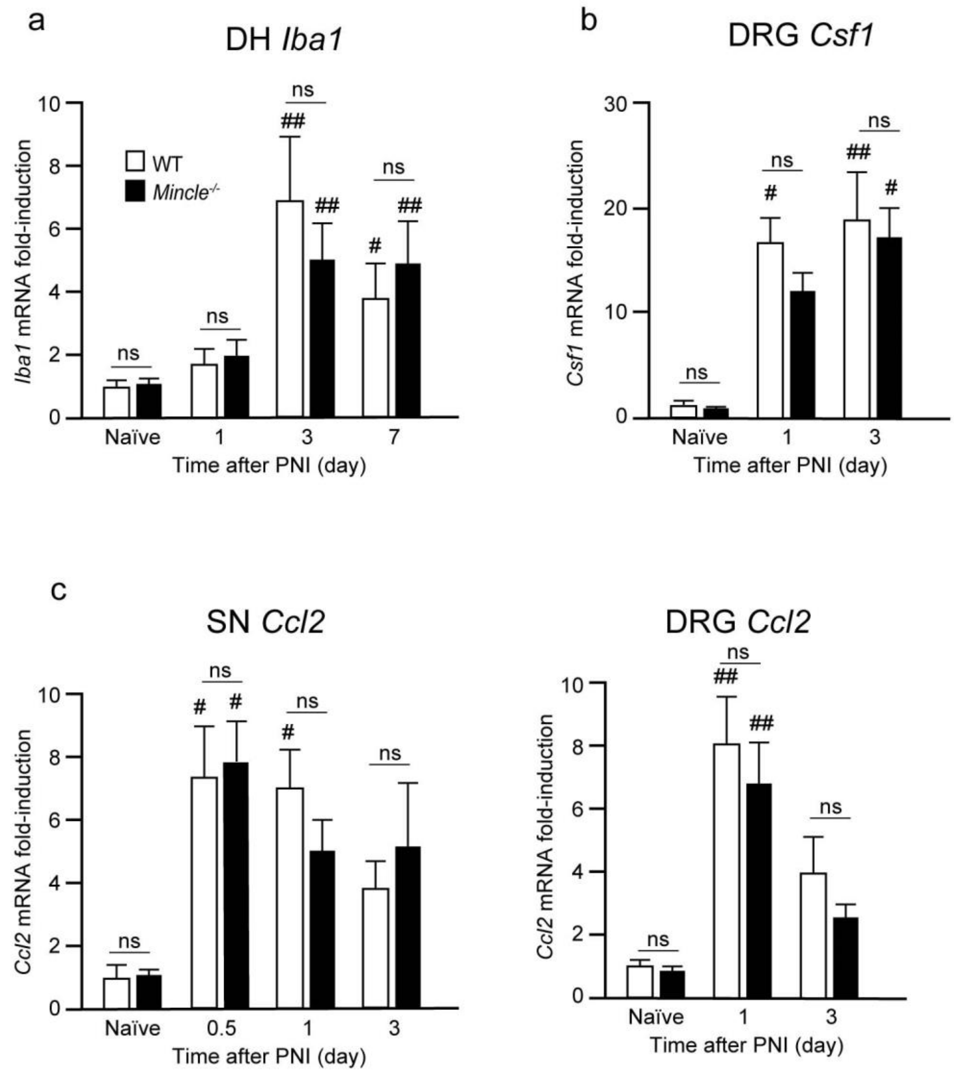

Supplementary Figure 2. Peripheral nerve injury (PNI)-induced mRNA expression of pain-related genes in the ipsilateral spinal nerve (SN), dorsal root ganglion (DRG) and spinal dorsal horn (DH) in WT and *Mincle*$^{-/-}$ mice. Each graph shows fold-induction of gene expression compared to naïve WT mice, for *Iba1* in the DH (a, n = 4 in each group), *Csf1* in the DRG (b, n = 3 in each group), and *Ccl2* in the SN and DRG (c, n = 3-6 in each group). Note that fold-induction of these genes was comparable between WT and *Mincle*$^{-/-}$ mice. Two-way ANOVA followed by Tukey's *post hoc* test, $^{\#}P < 0.05$, $^{\#\#}P < 0.01$ versus naïve controls. Values are represented as the mean ± SEM.

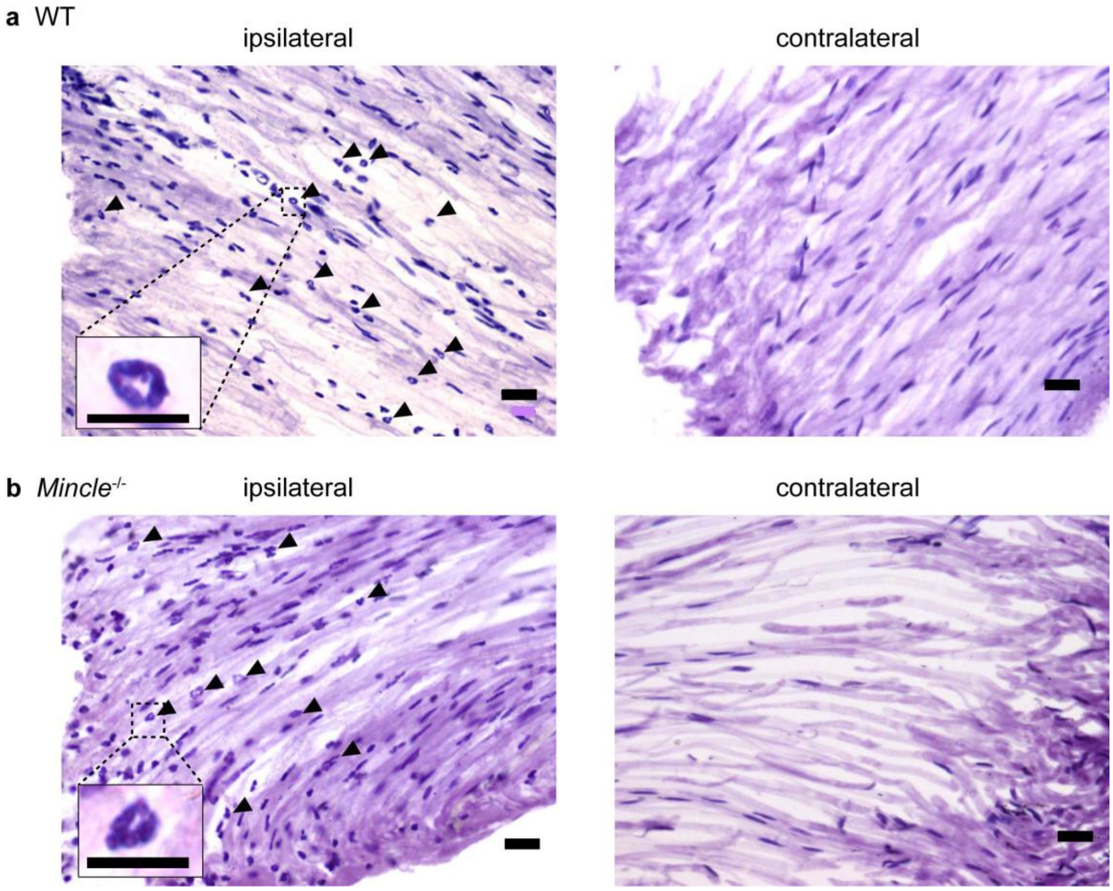

Supplementary Figure 3. PNI-induced leucocytes infiltration in injured SNs. Representative haematoxylin sections of SNs from WT (a) and *Mincle*$^{-/-}$ (b) mice 12 h after PNI (Scale bar, 20 μm). The insets show the representative morphology of infiltrated leucocytes at higher magnification (Scale bar, 10 μm). Arrow heads indicate infiltrated leucocytes.

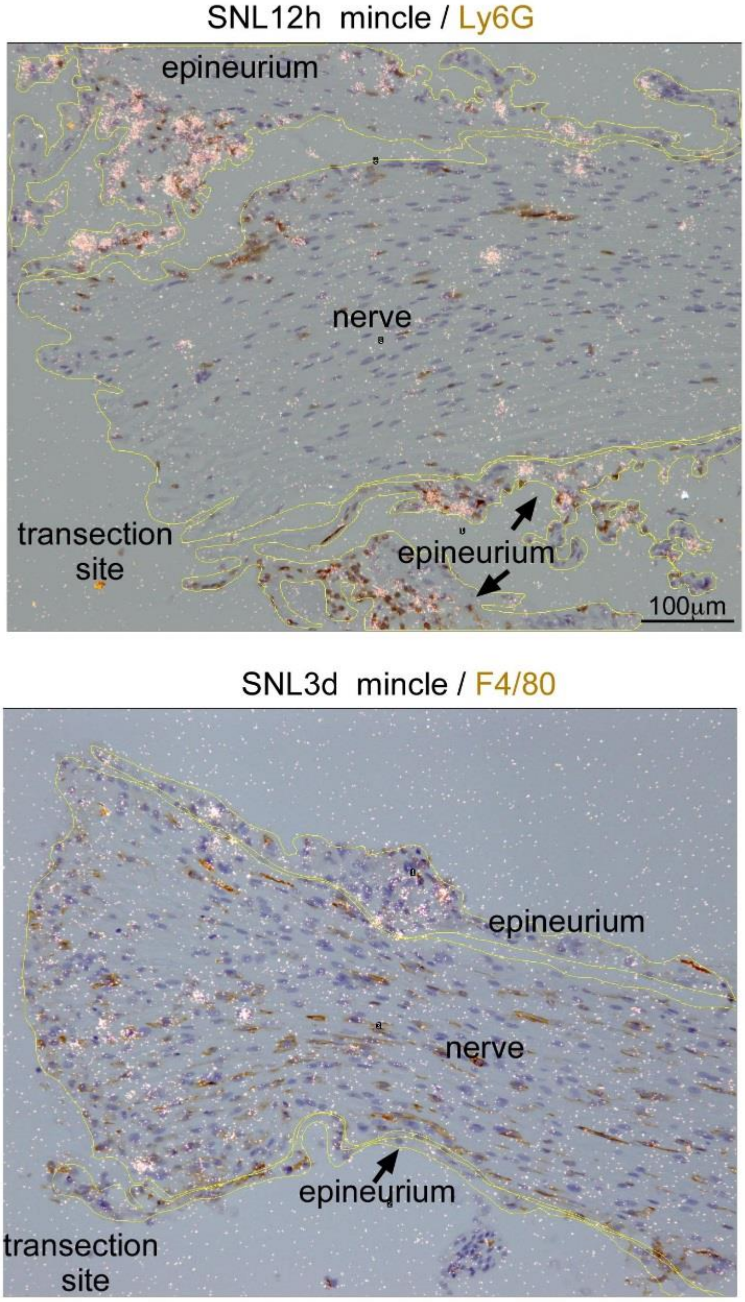

Supplementary Figure S4. PNI-induced *Mincle* mRNA expression in migrating neutrophils and macrophages/monocytes in the injured SN. Dark-right-field photomicrographs of combined ISHH for *Mincle* mRNA with immunostaining with Ly6G or F4/80 in the ipsilateral SN. Yellow border indicate that the measurement area of nerve or epineurium. Arrowheads indicate epineurium.

Supplementary table S1 The numbers, percentages, and densities of Ly6G$^+$ and/or Mincle$^+$ cells 12 h after nerve injury.

	Area measured (µm^2)	Number of cells counted			Percentage		Cell density (/10^4 µm^2)		
		Ly6G$^+$	Mincle$^+$	Ly6G$^+$Mincle$^+$	Ly6G$^+$/Mincle$^+$	Mincle$^+$/Ly6G$^+$	Ly6G$^+$	Mincle$^+$	Ly6G$^+$ Mincle$^+$
Nerve	3,121,398.3	560	146	80	54.8%	14.3%	1.79	0.47	0.26
Epineurium	920,510.4	1,115	679	476	70.1%	42.7%	12.11	7.38	5.17
Total	4,041,908.8	1,675	825	556	67.4%	33.2%	4.14	2.04	1.38

Data were obtained from 12 sections.

Supplementary table S2 The numbers, percentages, and densities of F4/80$^+$ and/or Mincle$^+$ cells 3 days after nerve injury.

	Area measured (µm^2)	Number of cells counted			Percentage		Cell density (/10^4 µm^2)		
		F4/80$^+$	Mincle$^+$	F4/80$^+$Mincle$^+$	F4/80$^+$/Mincle$^+$	Mincle$^+$/F4/80$^+$	F4/80$^+$	Mincle$^+$	F4/80$^+$Mincle$^+$
Nerve	3,286,389.0	969	249	113	45.4%	11.7%	2.95	0.76	0.34
Epineurium	590,917.1	330	86	27	31.4%	8.2%	5.58	1.46	0.46
Total	3,877,306.1	1,299	335	140	41.8%	10.8%	3.35	0.86	0.36

Data were obtained from 15 sections.
